# Prevalence, predictors and economic consequences of no-shows

**DOI:** 10.1186/s12913-015-1243-z

**Published:** 2016-01-14

**Authors:** Parviz Kheirkhah, Qianmei Feng, Lauren M. Travis, Shahriar Tavakoli-Tabasi, Amir Sharafkhaneh

**Affiliations:** 1 Office of Performance Improvement, University of Texas MD Anderson Cancer Center, Houston, TX USA; 2 Industrial Engineering Department, University of Houston, Houston, TX USA; 3 Audie L. Murphy VA Medical Center, San Antonio, TX USA; 4 Baylor College of Medicine, 2002 Holcombe Blvd., Houston, TX 77030 USA; 5 Michael E. DeBakey VA Medical Center, 2002 Holcombe Blvd., Houston, TX 77030 USA

**Keywords:** Health Care Use, Reminder system, Health care resource planning

## Abstract

**Background:**

Patients not attending to clinic appointments (no-show) significantly affects delivery, cost of care and resource planning. We aimed to evaluate the prevalence, predictors and economic consequences of patient no-shows.

**Method:**

This is a retrospective cohort study using administrative databases for fiscal years 1997–2008. We searched administrative databases for no-show frequency and cost at a large medical center. In addition, we estimated no-show rates and costs in another 10 regional hospitals. We studied no-show rates in primary care and various subspecialty settings over a 12-year period, the monthly and seasonal trends of no-shows, the effects of implementing a reminder system and the economic effects of missed appointments.

**Results:**

The mean no-show rate was 18.8 % (2.4 %) in 10 main clinics with highest occurring in subspecialist clinics. No-show rate in the women clinic was higher and the no-show rate in geriatric clinic was lower compared to general primary care clinic (PCP). The no-show rate remained at a high level despite its reduction by a centralized phone reminder (from 16.3 % down to 15.8 %). The average cost of no-show per patient was $196 in 2008.

**Conclusions:**

Our data indicates that no-show imposed a major burden on this health care system. Further, implementation of a reminder system only modestly reduced the no-show rate.

## Background

Healthcare systems consumed 16 % of the U.S. Gross Domestic Product (GDP) in 2007. Its share of GDP is projected to reach 19.5 % by 2017 [1]. To confront rising costs, limited resource capacities, and burgeoning demands facing U.S. healthcare providers, efficiently using clinical resources is critical. A major obstacle in cost-effective health care delivery and patient safety is no-show. Patient no-show is defined as a patient who does not appear for the scheduled appointment.

The extent and the cost of no-shows are widely studied. In a community hospital setting, Clark [2] reported an average no-show rate of 62 appointments per day and an estimated annual cost of $3 million. In a training hospital setting, Xakellis and Bennett [3] reported a 25 % no-show rate and a 31 % late arrival to appointments. Moore and colleagues [4] found that no-shows and cancellations represented 31.1 % of overall scheduled appointments among approximately 45,000 patients per year at a large family practice center with an estimated total annual revenue shortfall of 3 % to 14 %. Further, several studies found no-show rates of as low as 3 % and as high as 80 % [5–12].

Providers use different methods to reduce the patient no-show, including reminder procedures, penalization, and overbooking. The success of the methods is not clearly determined. Satiani and colleagues [13] reported that automated reminder systems did not significantly reduce the no-show rate. Hixon and colleague [14] found no difference in no-show rates when clinics use reminder systems to reduce no-show.

Delayed testing potentially puts patients in danger. Missed screening or patient no-show may results in delayed disease detection. Reducing no-show rates can diminish cost and improve quality of health care delivery.

The U.S. Veterans Health Administration (VHA) provides care to more than 6 million veterans. In the health care system the services provided, the decisions made, and the treatment selected is not affect by the patients’ income. For researchers, the VHA provides a unique opportunity to study issues related to the delivery of health care system. Users of VHA system receive almost all of their care from VHA and comprehensive nationwide databases are used to record all impatient and outpatient visits.

In this study, we investigated the extent of no-show and factors such as hospital size, gender, and age that may affect it. We also calculated consequences and examined the effect of implementing different methods on reducing no-show rates in a large tertiary VHA medical center in Houston with several satellite clinics in more remote areas. The study of no show in this population is unique as VHA is one of the largest government healthcare systems in USA with no penalty for no show.

## Methods

This is a retrospective review of information compiled in administrative databases for a 12-year period. This study was approved by the local institutional review board and local Veterans Affairs research and development (R&D) committee.

### Data collection

The Michael E. DeBakey VA Medical Center (MEDVAMC) at Houston, Texas, serves more than 76,000 Veterans in southeast Texas. Further, it operates four additional satellite outpatient clinics in areas remote to Houston.

Because of the large number of visits each year, in the first part of our study, we focused on the no-show data for the last 12 (fiscal year 1997–2008) years in 10 main clinics including Audiology, Cardiology, Dermatology, Eye Care, Gastroenterology, Mental Health, Orthopedics, Podiatry, Primary Care, and Urology. Each fiscal year starts on October of the preceding year and ends on last day of September of that year. In the second part of study, for statistical and economical analysis, all the no-show data between fiscal year 2006 to 2008 were extracted and analyzed.

### Statistical and economical analysis methods

This study focused on outpatient appointments. If a patient showed up for his or her appointment, the visit was classified as a “completed appointment (CA).” A Missed appointment (MA) was the categorization assigned where a patient did not come to the appointment. MAs are produced by several reasons including clinic cancellation, patient cancellation, or patient no- show. We define a no-show event as a patient that did not appear for the appointment or cancelled the appointment so late (usually on the day of the appointment) that scheduling another patient to that time slot was not feasible. The total number of no-show events is calculated as the sum of all no-shows. The no-show rate was calculated as the proportion of the total no-shows to the number of all appointments.

We used SAS9.2 (SAS, Inc., Cary, NC) for the analysis. We summarized descriptive statistics as mean and standard deviation and investigated the predictive value of various factors including demographic (age and gender) and clinic assignment characteristics (group vs. individualized appointment in mental health), hospital size, and appointment time-of-day or day-of-week on no-show rate. All the years in this study are fiscal years that start from October of the previous year and end in September of the desired year. All statistical hypothesis tests were conducted at the 5 % level of significance. We used Kolmogorov-Mirnov test for normality test and based on the result of normality test, a suitable statistical analysis (parameteric vs. nonparametric) was conducted. Also desirable two-way ANOVA’s were applied to study different factor effects.

For economic analysis, we used the average cost per encounter in MEDVAMC. The cost includes the total direct costs (supplies and fixed direct) and indirect costs. If the facility cannot fill a slot freed by a no-show patient, the cost to pay staff (physician, nurse, and other administrative staff) remains. To estimate the marginal cost of a no-show, we used the overall cost of visit per encounter because the majority of the cost per encounter consists of the staff costs. We were unable to consider some of the freed slots that were filled by walk-in patients because the data for each department were not available.

## Results

### No-show rates

During the study period, mean (SD) no-show rate was 18.8 % (2.4 %) in the 10 clinics. Gastro-intestinal (25.7 % (4.1 %)) and Audiology (12.6 % (2.0 %)) had the highest and the lowest no-show rates, respectively. Primary care had the highest number of visits (an average of 185,945) and consequently the highest total number of no-show patients (an average of 33,098 no-shows per year) per year.

### Cost of no-show

We reviewed the average actual cost per encounter in MEDVAMC and the corresponding national cost for 10 clinics in 2008. In general, the MEDVAMC has lower costs per encounter than the national average costs. The mean (SD) of actual cost per encounter in MEDVAMC was $167 ($67). Podiatry ($58) and Primary Care ($274) had the highest and the lowest actual cost per encounter, respectively.

Using actual cost per encounter and number of no-show in each clinic during the study period, we were able to estimate marginal cost of the no-shows. In fiscal year 2008, the estimate of the marginal cost of no-shows for the 10 clinics was $14.58 million while the lowest and highest marginal cost happened in 1997 and 2005 with $10.48 and $16.65 million, respectively. To have a better perspective of no-show rates and costs, we calculated the number of no-shows for all clinics during the last 3 fiscal years. Table 1 represents the no-show rates and costs for all clinics in MEDVAMC. The average cost of no-show per patient was $196 in 2008.

**Table 1:** Estimate of marginal cost of no-show for all clinics

Year	# Appointment	# No-show	No-show rate	Cost ($M)
2006	979,981	158,751	16.2 %	27.36
2007	921,221	129,324	14.0 %	23.98
2008	1,028,300	146,358	14.2 %	28.66

### Effects of important factors on no-show rates

#### Effect of gender on no-show

The women clinic at MEDVAMC provides primary care to female veterans. To investigate the effect of gender on no-show rates, we compared the no-show rate of the women clinic and that of the primary care clinic (PCP), because patients in women clinic are exclusively women while overwhelming majority of patients in PCP are men. We reviewed no-show rates for 60 months from fiscal year 2004 to 2008. The no-show rates data in PCP is not normally distributed (p < 0.01). Therefore, we applied sign-test to compare no-show rates in the two clinics. Women clinic had higher no-show rate than PCP (p <0.001). However, the no-show rate difference between the women and the PCPs disappeared at last two years of the study period.

#### Effect of age group on no-show

To identify the effect of age on the no-show rate, we compared the no-show rate of geriatric PCP and that of the general PCP. The geriatric PCP delivers service to senior patients with age 65 or higher. We compared the no-show data in geriatric PCP and PCP for 60 months from 2004 to 2008. Using the sign-test, geriatric clinic had less no-show than PCP (p < 0.001).

#### Effect of group treatment in mental health on no-show

To investigate the effect of group treatment on no-show rate, we studied the no-show data in mental health individual and group clinic visits. We extracted the no-show rates of both clinic visit types for 36 months from fiscal year 2006 to 2008. The sign-test showed that the group had more no-show than the individual clinic visit (p < 0.001).

#### Effect of hospital size on no-show

**Fig. 1:**
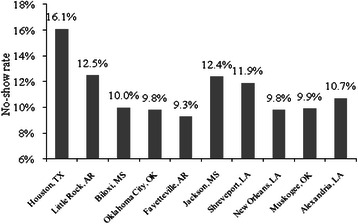
No-show rate for 10 main clinics in different VA hospitals at VISN16 in 2008

The size of a hospital may have a significant effect on the no-show rate, which can be measured by the number of patient visits per year. Among the 10 hospitals in South Central VA Health Care Network or VISN 16, the largest VA hospital is the MEDVAMC with 76,745 patients and the smallest one is the VA hospital in Alexandria, LA with 24,541 patients (Fiscal Year or FY 2008). The no-show rates shown in Fig. 1 are calculated based on the 10 main clinics for the 10 hospitals in fiscal year 2008. It can be observed that the MEDVAMC had the largest no-show rate in 2008 among the 10 hospitals in Veteran Integrated Service Network region 16 (VISN 16).

To examine the effects of different hospitals and different clinics on no-show rate, we conducted a two-way ANOVA test without replication for no-show rates among 10 different hospitals and the 10 clinics in each hospital for fiscal year 2008. The no-show rate differed significantly among the VA hospitals in VISN 16 and among different clinics in the hospitals (p value for both <0.001).

#### Effect of appointment time on no-show

**Fig. 2:**
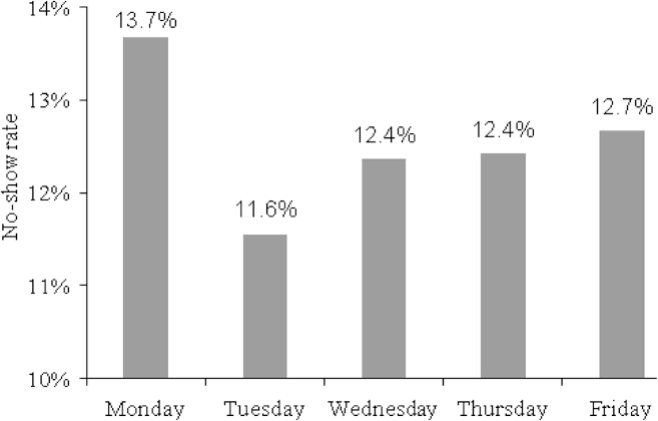
No-show rate in each day for 10 main clinics in VA hospital at Houston in 2008

To study the effect of appointment times on the no-show rates, 10 random weeks in 2008 were selected and the no-shows rates were calculated for the 10 clinics for the five weekdays. As shown in Fig. 2, the no-show rate on Mondays was the highest, and then it dropped on Tuesdays followed by a smooth increase until Fridays. A two-way ANOVA test showed that the day of a week has a small but statistically significant effect on no-show (p = 0.03) and the type of clinic has a significant effect on no-show (p-value < 0.001).

**Fig. 3:**
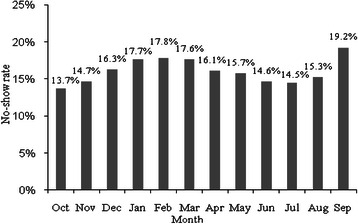
No-show rate in each month for 10 main clinics in VA hospital at Houston (2008)

To study the effect of the month of an appointment on no-show rate, we calculated the rate of no-shows in each month for all the 10 clinics in fiscal year 2008 at MEDVAMC. As shown in Fig. 3, the first 3 months in 2008 had the highest no-show rates with an average of 17.7 %. Then it decreased until September when it increased suddenly and reached to 19.2 % as the highest rate in the year. A two-way ANOVA test for month and clinic showed that both the month of appointment and the type of clinic have significant effects on no-show (p-values < 0.001).

### Effectiveness of different methods to reduce no-show rates

MEDVAMC implemented methods to reduce the no-show rate including a reminder-letter system and a centralized phone system.

#### Effectiveness of a reminder-letter system

**Fig. 4:**
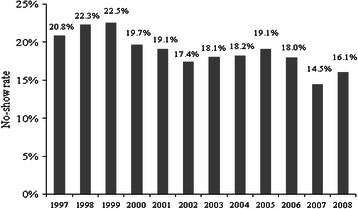
No-show rates in 10 main clinics in VA hospital at Houston

A reminder-letter system is used to remind the patients about their appointment times since 2004. Figure 4 displays the no-show rates for all the 10 clinics from 1997 to 2008. Although the rates of no-show increased unexpectedly in 2005 (from 18.2 % in 2004 to 19.1 % in 2005), the average of no-show rates for 4 years before applying the reminder system was 18.17 % while the mean decreased to 16.96 % for 4 consecutive years after applying the reminder system.

To further study the effect of the reminder system on no-show while adjusting for the effect of the months, we designed a test to compare the no-show rates in all the 10 clinics for 12 months in 2004 with the same months and clinics in 2005. Using sign test, the no-show rate did not change after implementing the reminder system (p = 0.70).

#### Effectiveness of a centralized phone system

To reduce no-show rates, the MEDVAMC applied a new centralized phone system in October 2008. Using the centralized phone system, only one toll free number is used to handle scheduling of appointments for all clinics.

**Fig. 5:**
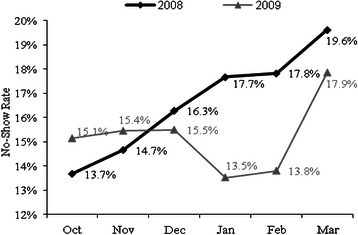
No-show rate in 10 main clinics before and after applying new phone system

To study the effect of the new phone system, all no-show data were pulled out for the 10 clinics from October 2008 to March 2009, a sample of 60 no-show rates. To remove the monthly effect of appointment time, we compared no-show in the six months with the same months in the previous year, i.e., October 2007-March 2008. Figure 5 represents the trend of no-show rates before and after applying the new phone system. Using sign-test, the no-show rates decreased significantly during the first 6 months of applying the new phone system (p value = 0.03). The average no-show rate in the 10 clinics decreased from 16.3 % in 2008 to 15.2 % in 2009.

## Discussion

In this paper, we investigated the presence, extent and economic consequences of patient no-show and explored the factors that may modulate no-show rates, and the effect of implementing a reminder system to reduce no-show rates. This study is one of the first comprehensive studies on no-show rates at the VHA system. Our data showed that no-show rate was high with significant economic cost. Further, our study identified various factors including gender, age, visit related specialty, type of the visit (group vs. individual), day of the week, and the month of the visit as potential factors associated with no-show. Our study is unique from several aspects. In contrast to other no show studies, our study encompasses the largest number of patients. VHA is a government run health care system with minimal or no cost to eligible users. Thus, our data evaluates no show in a health care system that is not costly to the user. Further, the VHA patients are mostly older, men and with more comorbid conditions that may affect the use of health care and no show behavior.

The no-shows is a prevalent problem in many health care systems [3–11]. Our data confirms the finding in the VHA system. Our data indicated that women may have higher no-show compared to men in the studied health care setting. This finding is unique and needs to be confirmed in another health care setting. It is likely that during the study period, women used the Women Clinic for women related health care rather than general primary care.

The data showed that geriatric patients had more no-show compared to general primary care patients. Potential factors causing higher no-show in elderly may include age, health status, and retirement status and difficulty with transportation.

Our data showed that the no-show rate of the group treatment in mental health is higher than that of the individual treatment. Potential factors causing a higher no-show in group treatment may include unwillingness of patients to attend and discuss their problems in front of the others (lack of privacy) and different psychiatric conditions treated in group versus individual visits. This finding needs to be confirmed.

The clinic type in each VHA hospital in VISN 16 is a significant factor on no-show rates, and VHA hospitals with subspecialty healthcare services have a higher no-show rate than specialty healthcare providers. Subspecialty clinics provide care to patients referred from various smaller peripheral facilities. Thus, the referred patients may require traveling longer distance for a subspecialty visit compared to their local PCP visit.

The data indicated that the day-of-week and the day-of-month of the appointment affect no-show. Therefore, the no-show rate of each patient for an appointment is a function of appointment time and it is not fixed during the year. This finding is very important as any intervention aimed at reducing the no-show should include the effects of day of the week and month of the appointment.

The huge cost of no-shows combining with other undesirable consequences of no-show motivated the management to use any method to decrease the no-show rate, which can decrease the overall cost of health care tremendously. The results showed that the current implemented reminder system had modest effect on no-show.

## Conclusions

Our findings for the effects of different factors on the no-show rates can be used for the prediction of no-show. Our study clearly showed that many factors affected no-show, including age, gender, type of clinic, time of appointment (day and month), distance, employment status, and patient health status. Therefore, any promising methodology to predict and reduce no-show should consider and examine the effect of these factors on prediction model.

## Abbreviations

PCP: Primary Care Clinic
GDP: Gross Domestic Product
VHA: Veterans Health Administration
R&D: Research and Development
MEDVAMC: Micahle E. DeBakey VA Medical Center
CA: Completed Appointment
MA: Missed Appointment
VISN: VA Health Care Network
FY: Fiscal Year
